# Immunoglobulin G subclass switching impacts sensitivity of an immunoassay targeting *Francisella tularensis* lipopolysaccharide

**DOI:** 10.1371/journal.pone.0195308

**Published:** 2018-04-09

**Authors:** Teerapat Nualnoi, Adam Kirosingh, Kaitlin Basallo, Derrick Hau, Marcellene A. Gates-Hollingsworth, Peter Thorkildson, Reva B. Crump, Dana E. Reed, Sujata Pandit, David P. AuCoin

**Affiliations:** Department of Microbiology and Immunology, University of Nevada, Reno School of Medicine, Reno, Nevada, United States of America; New York State Department of Health, UNITED STATES

## Abstract

The CDC Tier 1 select agent *Francisella tularensis* is a small, Gram-negative bacterium and the causative agent of tularemia, a potentially life-threatening infection endemic in the United States, Europe and Asia. Currently, there is no licensed vaccine or rapid point-of-care diagnostic test for tularemia. The purpose of this research was to develop monoclonal antibodies (mAbs) specific to the *F*. *tularensis* surface-expressed lipopolysaccharide (LPS) for a potential use in a rapid diagnostic test. Our initial antigen capture ELISA was developed using murine IgG3 mAb 1A4. Due to the low sensitivity of the initial assay, IgG subclass switching, which is known to have an effect on the functional affinity of a mAb, was exploited for the purpose of enhancing assay sensitivity. The ELISA developed using the IgG1 or IgG2b mAbs from the subclass-switch family of 1A4 IgG3 yielded improved assay sensitivity. However, surface plasmon resonance (SPR) demonstrated that the functional affinity was decreased as a result of subclass switching. Further investigation using direct ELISA revealed the potential self-association of 1A4 IgG3, which could explain the higher functional affinity and higher assay background seen with this mAb. Additionally, the higher assay background was found to negatively affect assay sensitivity. Thus, enhancement of the assay sensitivity by subclass switching is likely due to the decrease in assay background, simply by avoiding the self-association of IgG3.

## Introduction

*Francisella tularensis* is a Gram-negative, facultative intracellular pathogen that causes the zoonotic infection tularemia [1]. *F*. *tularensis* can infect more than 200 species of organisms such as mice, rats, squirrels, hares, rabbits, ticks, deer flies, and mosquitos, indicating that the bacterium has a remarkably broad range of reservoir hosts and vectors [1,2]. The bacterium has also been recognized as one of the most infectious pathogens, requiring as low as one colony-forming unit (CFU) of the organism to cause infection via the respiratory route [3]. Due to its extreme infectivity and ability to cause an aerosol infection, *F*. *tularensis* has been categorized as a Tier 1 select agent by the Centers for Disease Control and Prevention (CDC), emphasizing concerns about its potential use as a biological weapon [4–6].

*F*. *tularensis* can be subdivided into four subspecies or biovars (*tularensis*, *holarctica*, *novicida* and *mediasiatica*), but only subsp. *tularensis* and *holarctica* significantly impact human health [7]. Subsp. *tularensis* (type A) is the most virulent and is exclusively isolated in North America [8]. As an example, SCHU S4 is a *tularensis* subsp. strain that is commonly used in biosafety level 3 (BSL-3) laboratories [9]. Subsp. *holarctica* (type B) causes a milder infection compared to subsp. *tularensis*, but affects wider geographical areas of the northern hemisphere [8]. The live vaccine strain (LVS), an attenuated strain from subsp. *holarctica*, is widely used in tularemia research laboratories as a *F*. *tularensis* surrogate because it can be handled safely in a BSL-2 environment. However, the Food and Drug Administration (FDA) has not approved LVS for use as a human vaccine [9].

Tularemia is an acute infection with an average incubation period of 3–5 days. Early clinical presentations of the infection are nonspecific flu-like symptoms, making it difficult to diagnose, especially in areas where *F*. *tularensis* is uncommon [10]. Currently, bacterial culture remains the gold standard for diagnosis of tularemia [11]. Other serological methods such as enzyme-linked immunosorbent assay (ELISA) and agglutination tests targeting antibody against *F*. *tularensis* have also been used to assist in tularemia diagnosis [12,13]. However, the culture technique is time-consuming, and antibody takes about two weeks to appear in serum; as a consequence, an appropriate treatment is likely to be delayed [14]. Tularemia can be treated with antibiotics such as streptomycin, doxycycline and ciprofloxacin, but the mortality rate can be as high as 30–60% if antibiotic administration is delayed or not provided, emphasizing the need for a rapid diagnostic that can be performed at the point-of-care (POC) [15,16].

Lipopolysaccharide (LPS) is an outer membrane structure expressed by most Gram-negative bacteria, including *F*. *tularensis*. It consists of three major components: lipid A, core-oligosaccharide and repeating units of *O*-antigen [9]. Like many other Gram-negative bacterial pathogens, *F*. *tularensis* LPS has been recognized as a virulence factor and a protective antigen [9]. Additionally, a study from our laboratory revealed that LPS appears to be shed into serum during *F*. *tularensis* infection, suggesting that LPS is a potential diagnostic target for tularemia [17]. This finding encouraged us to develop a POC immunodiagnostic targeting *F*. *tularensis* LPS antigen.

Initially, an antigen capture immunoassay was developed using LPS-specific murine immunoglobulin G3 (IgG3) monoclonal antibody (mAb). However, the assay had lower sensitivity than other immunoassays that have been reported previously [18,19]. Recognizing that binding affinity of a mAb has a major impact on sensitivity of an assay [20], we hypothesized that the assay sensitivity could be improved by employing a mAb with higher binding affinity.

It has been long understood that specificity and affinity of an antibody is defined by the amino acid sequence of the antigen binding regions, located on the variable domains of an antibody. Accordingly, murine IgG3, IgG1, IgG2b and IgG2a that contain identical variable regions are believed to possess the same specificity and affinity. However, recently this paradigm has been challenged by a number of studies [21–25]. In one example, murine IgG1 mAb to the glucuronoxylomannan (GXM) capsule of *Cryptococcus neoformans* was found to have higher affinity than the IgG3 bearing the same antigen binding sequences [24]. In our current study, we investigated whether or not subclass switch could yield an increased binding affinity from the parental murine IgG3 specific to *F*. *tularensis* LPS, and improve sensitivity of the resulting antigen capture immunoassay as a consequence.

## Materials and methods

### Bacteria

The heat-inactivated *F*. *tularensis* LVS used for immunization and Western blotting was received from the laboratory of Dr. Terry Wu (University of New Mexico Health Sciences Center). Other bacteria or purified LPS used for Western blotting were collected from various sources. Inactivated *F*. *tularensis* SCHU S4 (NR-15753) and SCHU S4 ΔwbtI (NR-15754) were obtained from BEI Resources (Manassas, VA). Inactivated *F*. *tularensis* subsp. *holarctica* (FRAN-012), subsp. *novicida* (FRAN-003), *Francisella philomiragia* (FRAN-017) were received from the Department of Defense Critical Reagents Program (Frederick, MD). Purified *F*. *tularensis* LVS LPS (NR-2627) was also obtained from BEI Resources. Purified LPS from *Burkholderia pseudomallei* and *Escherichia coli* were a courtesy from Dr. Paul Brett and Dr. Mary Burtnick, Department of Microbiology and Immunology, University of South Alabama.

### Immunization of mice and production of mAbs

Female, 8-week old, BALB/c mice were immunized with 2 x 10^8^ colony forming units (CFU) of heat-inactivated *F*. *tularensis* LVS in 200 μL of Dulbecco’s phosphate-buffered saline (dPBS) via intraperitoneal injection. Antibody titers were monitored using an indirect ELISA. Three days prior to splenectomy, the mice were intraperitoneally injected with 2 x 10^8^ CFU of the inactivated bacterium. Hybridoma cell lines were generated using standard hybridoma techniques [26]. As a result, the hybridoma clone producing murine IgG3 specific to *F*. *tularensis* LVS LPS, 1A4 IgG3, was isolated. For large-scale production of the mAb, the hybridoma clone was propagated in Integra CL 1000 culture flasks (Integra Biosciences, Hudson, NH), antibody-rich supernatant was collected, and the mAb was purified using protein A affinity column chromatography.

IgG1 and IgG2b subclass variants of mAb 1A4 (1A4 IgG1 and 1A4 IgG2b) were sequentially isolated from parental 1A4 IgG3 that spontaneously underwent subclass switch using a modified method of sib selection as previously described [27]. After the hybridoma clones producing 1A4 IgG1 and 1A4 IgG2b were obtained, the mAbs were produced and purified using the same method described above.

DNA sequencing was performed to verify that the variable regions of all three subclass variants of the mAb are identical. Briefly, total RNA was isolated from hybridoma cell lines using Qiagen RNeasy Mini Kit (Qiagen, Hilden, Germany). cDNA libraries then were prepared using an oligo(dT) primer and SMART template-switching oligonucleotide [28]. Subsequent PCR reactions and sequencing were performed using heavy and light chain specific primers from Mouse Ig-Primer Set (Novagen, Madison, WI) and those described by Fukui et al. [29]. The sequences of variable regions including framework regions (FR) 1, 2 and 3 and complementarity-determining regions (CDR) 1, 2 and 3 were assigned and aligned using IMGT/V-Quest (http://www.imgt.org) [30].

### Ethics statement

The use of laboratory animals in this study was approved by the University of Nevada, Reno Institutional Animal Care and Use Committee (protocol number 00024). All work with animals at the University of Nevada, Reno is performed in conjunction with the Office of Lab Animal Medicine, which adheres to the National Institutes of Health Office of Laboratory Animal Welfare (OLAW) policies and laws (assurance number A3500-01).

### Western blotting

Samples including inactivated *F*. *tularensis* SCHU S4, SCHU S4 ΔwbtI, LVS, *F*. *tularensis* subsp. *holarctica*, subsp. *novicida*, *Francisella philomiragia*, purified *Burkholderia pseudomallei* LPS and *Escherichia coli* LPS were mixed with SDS-PAGE sample buffer and boiled for 10 min. The samples then were incubated with proteinase K at 60°C for 1 hour before separation with 12% TGX precast gels (Bio-Rad, Hercules, CA). Western blotting was performed with mini-nitrocellulose transfer packs and a Trans-Blot Turbo transfer system (Bio-Rad). The membranes were blocked with 5% skim milk in TBS-Tween (TBS-T: 50 mM Tris, 150 mM NaCl, 0.1% Tween 20, pH 7.6) at 4°C overnight, followed by incubation with 1 μg/mL of mAb 1A4 or mAb FB11 (a commercially available *F*. *tularensis* LVS LPS-specific mAb, Novus Biologicals, Littleton, CO) for 90 min at room temperature. After washing with TBS-T, the membranes were incubated with an anti-mouse IgG horseradish peroxidase (HRP) conjugate (Southern Biotech, Birmingham, AL) for 60 min at room temperature to facilitate detection. The final development was carried out using Pierce ECL Western Blotting Substrate (Pierce Biotechnology, Rockford, IL) and a ChemiDoc XRS imaging system (Bio-Rad). A replica gel was stained with Pro-Q Emerald 300 LPS gel stain kit (Molecular Probes, Eugene, OR) using the protocol described by the manufacturer to demonstrate the presence of LPS in these samples.

### Antigen capture immunoassay (sandwich ELISA)

Microtiter plates were coated overnight at room temperature (RT) with mAb 1A4 IgG3, IgG1 or IgG2b in PBS with the concentrations pre-optimized by checkerboard ELISA (S1 Table). After washing with PBS-Tween (PBS-T: PBS containing 0.05% Tween 20), the plates were blocked with a blocking solution (PBS containing 0.5% Tween 20 and 5% non-fat milk) at 37°C for 1 hour, then washed with the blocking solution. A two-fold serial dilution series (starting with 30 ng/mL) of purified *F*. *tularensis* LVS LPS in blocking solution was added to the plates and incubated at RT for 1.5 hours. After washing with the blocking solution, the plates were incubated at RT for 1 hour with mAb 1A4 IgG3, IgG1 or IgG2b labeled with HRP. The concentrations of HRP-conjugated mAbs are shown in S1 Table. HRP conjugation was carried out using the EZ-Link Plus activated peroxidase kit (Pierce Biotechnology). Following a wash with PBS-T, the plates were incubated with tetramethylbenzidine (TMB) substrate (KPL, Gaithersburg, MD) for 30 min. The reaction was stopped with 1 M H_3_PO_4_, and the optical density at 450 nm (OD_450_) was read using the Emax^®^ microplate reader (Molecular Devices, Sunnyvale, CA). The limit of detection (LOD, in ng/mL) of the assays then was determined using SoftmaxPro (Molecular Devices). To determine the LOD in CFU/mL, the entire experiment was repeated, except for the serial dilution of the purified *F*. *tularensis* LPS was replaced by a serial dilution series (starting at 10^6^ CFU/mL) of the inactivated *F*. *tularensis* LVS bacterial suspension. In this study, we defined the LOD as the concentration of LPS (or bacterial density) that provided a twofold increase of OD_450_ above background.

### Competition ELISA

The LPS binding affinities of each 1A4 subclass variant were compared by a modified method of competition ELISA described by Fránek et al. [31]. Microtiter plates were coated overnight with 10 ng/mL of purified *F*. *tularensis* LVS LPS in PBS at RT, washed with PBS-T, blocked with the blocking solution at 37°C for 1 hour, and washed again with the blocking solution. To generate dose-response curves for the 1A4 subclass variants, each variant was mixed with 1A4 IgG1 HRP conjugate in microcentrifuge tubes. The concentration of 1A4 IgG1 HRP conjugate was fixed at saturation (3 μg/mL) while the concentrations of each unlabeled 1A4 subclass variant were varied by two-fold serial dilution. The mixtures then were added to the plates, and incubated for 1.5 hours. After the incubation, the plates were washed with PBS-T, and incubated with TMB substrate for 30 min. The reaction was stopped with 1 M H_3_PO_4_, and the OD_450_ was read. Percent inhibition was calculated by % inhibition = [(OD_450_ of 1A4 IgG1 HRP alone–OD_450_ of 1A4 IgG1 HRP plus unlabeled mAb)/ OD_450_ of 1A4 IgG1 HRP alone] x 100. The dose-response curves (% inhibition *vs* mAb concentration) for each 1A4 subclass variant then were created. IC_50_ values (the concentration of each 1A4 subclass variant that inhibits the binding of 1A4 IgG1 HRP conjugate by half) were calculated from the sigmoidal dose-response curve model using SigmaPlot 13.0 (Systat Software Inc., San Jose, CA).

### Direct ELISA

Direct ELISA was used to investigate potential antibody-antibody interactions. Microtiter plates were coated overnight at RT with 2 μg/mL of 1A4 IgG3, IgG1 or IgG2b mAb, washed with PBS-T, and then blocked with blocking solution at 37°C for 1 hour. After washing with blocking solution, the plates were incubated at RT for 1.5 hours with two-fold serial dilutions of 1A4 IgG3, IgG1 or IgG2b mAb HRP conjugate. After the incubation, the plates were washed and developed, and OD_450_ was measured as described above.

### Surface plasmon resonance

All surface plasmon resonance (SPR) experiments in this study were performed using a BIAcore X100 instrument (GE Healthcare). All analysis was conducted in HBS-EP+ (10 mM HEPES, 150 mM NaCl, 3 mM EDTA and 0.05% v/v Surfactant P20, GE Healthcare) as a running buffer and diluent. The steady-state affinity (*K*_D_) was calculated using a steady-state model in BIAevaluation software version 2.0.1 (GE Healthcare). Accuracy of the model fitting was assessed by χ^2^ parameter.

SPR experiments were carried out using two different assay configurations. For the first configuration, biotinylated *F*. *tularensis* LVS LPS was immobilized on a streptavidin (SA)-coated sensor chip (GE Healthcare, Piscataway, NJ); a second flow cell surface was left unmodified for reference subtraction. The biotinylated LPS was prepared using EZ-Link Sulfo-NHS-LC-Biotin (Pierce Biotechnology). To generate sensorgrams, different concentrations of 1A4 IgG3 (10.4–333 nM), 1A4 IgG1 (1.7–8 μM) or 1A4 IgG2b (0.1–3.3 μM) mAbs were injected over the sensor chip surface with a flow rate of 30 μL/min for 180 seconds, followed by passive dissociation for 300 seconds (or 600 sec for 1A4 IgG3 mAb). Between each cycle, the sensor chip surface was regenerated with 3M MgCl_2_ (IgG1 and IgG2b) or 10 mM NaOH and 0.5% SDS (1A4 IgG3).

For the second assay configuration, anti-mouse antibody was covalently immobilized via amine-coupling reaction on a CM5 sensor chip (GE Healthcare). The immobilization and assay were performed using a mouse antibody capture kit (GE Healthcare) with the instructions provided by the manufacturer. Each analysis cycle began with antibody capture by injecting 1 μg/mL of mAb 1A4 IgG3, IgG1 or IgG2b over the sensor chip surface at a flow rate of 30 μL/min for 60 seconds; a flow cell was left un-injected for reference subtraction. LPS at various concentrations (60–8,000 nM) then was injected over the chip surface at a flow rate of 30 μL/min for 120 seconds, followed by passive dissociation for 300 seconds. Between each cycle, 10 mM glycine HCl (pH 1.7) was injected for 60 seconds to regenerate the chip surface.

## Results

### Specificity of 1A4 mAb

**Fig 1:**
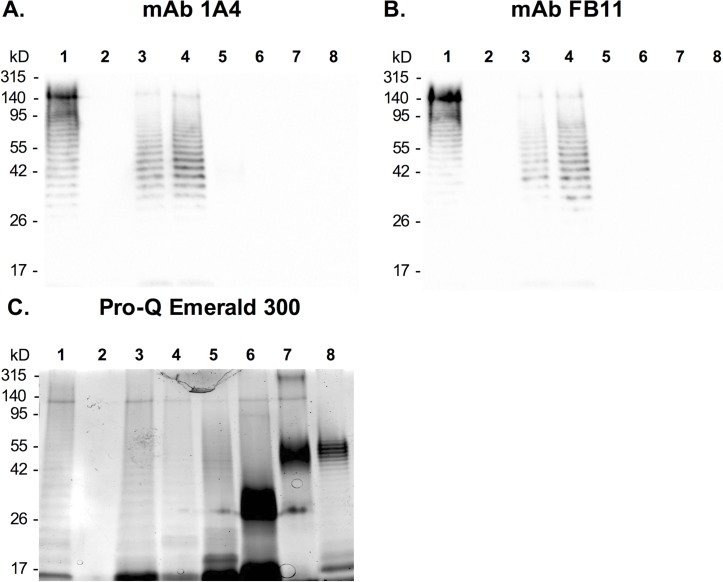
Specificity of mAbs 1A4 and FB11. Inactivated *F*. *tularensis* SCHU S4 (lane 1), SCHU S4 ΔwbtI (lacks *O*-antigen, lane 2), LVS (lane 3), *F*. *tularensis* subsp. *holarctica* (lane 4), subsp. *novicida* (lane 5), *F*. *philomiragia* (lane 6), purified *B*. *pseudomallei* LPS (lane 7) and *E*. *coli* LPS (lane 8) were separated on 12% SDS-PAGE gels and blotted onto nitrocellulose membranes. The membranes then were probed with mAbs 1A4 IgG3 (**Panel A**) and FB11 (**Panel B**). The existence of LPS in these samples was demonstrated by Pro-Q emerald 300 LPS staining (**Panel C**).

*F*. *tularensis* LPS-specific IgG3 mAb 1A4 was produced from mice immunized with heat-inactivated *F*. *tularensis* LVS bacteria. Specificity of mAb 1A4 was evaluated by Western blotting in comparison with a commercially available *F*. *tularensis* LPS mAb FB11. The results showed that mAb 1A4 reacted with purified LPS from *F*. *tularensis* LVS in a similar ladder-banding pattern as seen with mAb FB11, suggesting that mAb 1A4 recognizes the LPS structure (Fig 1). mAb 1A4 also reacted with *F*. *tularensis* strains SCHU S4 and LVS, and *F*. *tularensis* subsp. *holarctica*, but showed no reactivity to *F*. *tularensis* subsp. *novicida*, *F*. *philomiragia*, *Burkholderia pseudomallei* or *Escherichia*. *coli*. These results indicated that mAb 1A4 is specific to LPS from *F*. *tularensis* subsp. *tularensis* and *holarctica*, with no cross-reactivity to *novicida* subspecies or certain other Gram-negative bacteria. The results also showed that mAb 1A4 has no reactivity with *F*. *tularensis* SCHU S4 ΔwbtI (lacking *O*-antigen), confirming that mAb 1A4 recognizes the *O*-antigen moiety of LPS.

### Verification of variable region of mAb 1A4 subclass family

**Fig 2:**
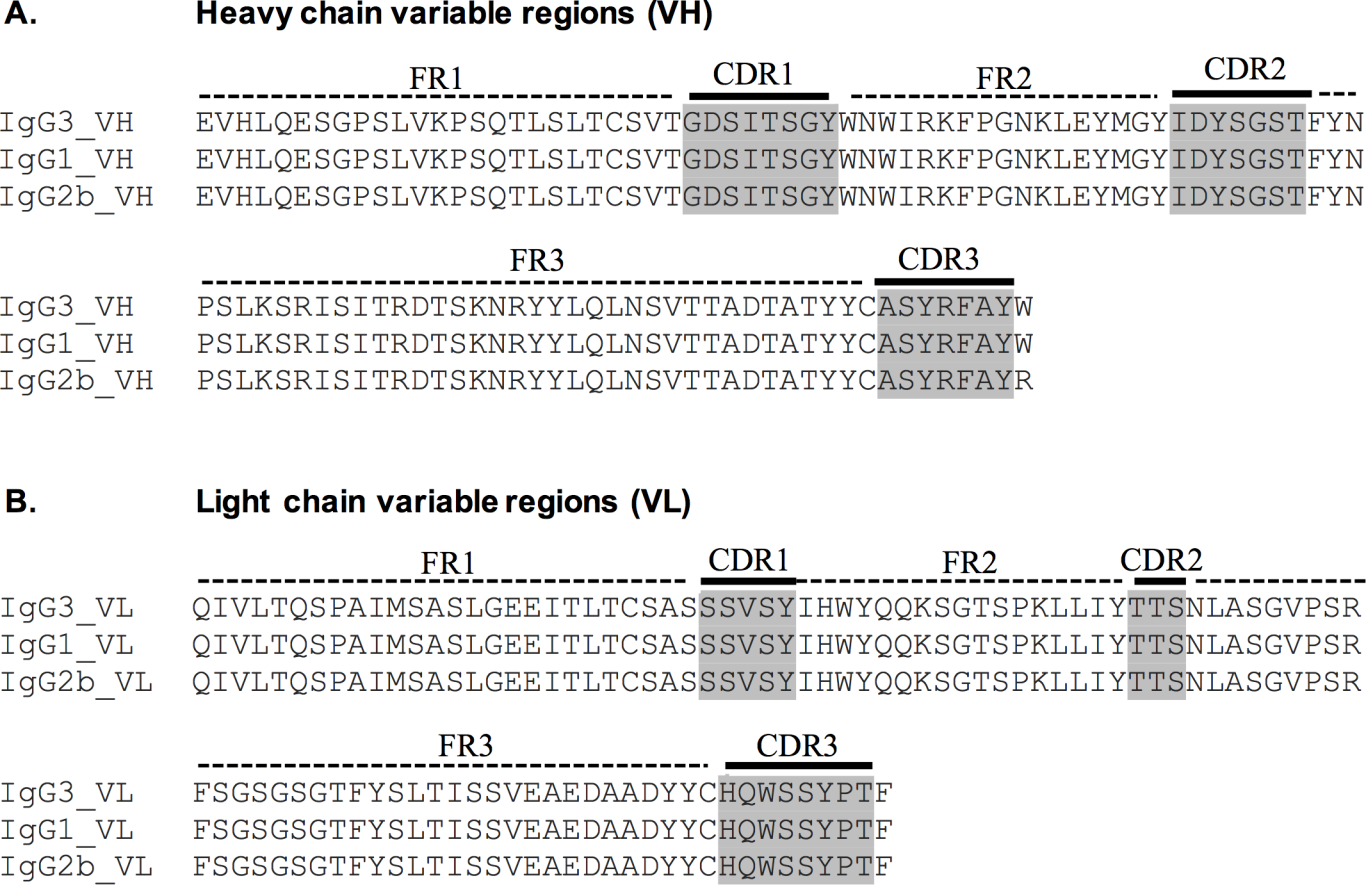
Sequence alignments of mAb 1A4 IgG3, IgG1 and IgG2b variable regions. The nucleotide sequences of mAbs 1A4 IgG3, IgG1 and IgG2b variable regions were analyzed using IMGT/V-Quest (www.imgt.org). The resulting amino acid sequences of heavy chain (**Panel A**) and light chain (**Panel B**) framework regions (FR) 1, 2, and 3 and complementarity-determining regions (CDR) 1, 2, and 3 are displayed. The grey highlights indicate the amino acid residues of CDRs.

To investigate the contribution of antibody constant region to assay sensitivity, mAbs 1A4 IgG1 and IgG2b were isolated from the parental IgG3 using a modified method of sib selection described previously [27]. cDNA cloning and sequencing were performed to verify variable regions of the 1A4 subclass variants. The results indicated that heavy chain and light chain CDRs of 1A4 IgG3, IgG1 and IgG2b are identical, confirming that they bear the same variable regions (Fig 2).

### Limit of detection of antigen-capture immunoassay

Antigen-capture immunoassays for detection of *F*. *tularensis* LPS were developed by using different combinations of the 1A4 subclass variants. The LOD (ng/mL and CFU/mL) of each mAb combination was determined and is summarized in Tables 1 and 2. The background readings (OD_450_ in the absence of purified LPS or inactivated bacterial cells), which were used for the LOD calculations, are presented in S2 and S3 Tables. We found that the LODs determined by using purified LPS (Table 1) and inactivated bacteria (Table 2) were consistent, in that the results showed that using IgG3 as both capture and detector mAbs simultaneously gave the poorest assay sensitivity, with a LOD of 2.4 ng LPS/mL or 1.0 x 10^5^ CFU/mL (Tables 1 and 2, bold). Sensitivity of the assay was improved (up to 4 fold) when the detector mAb was switched from IgG3 to IgG1 or IgG2b. In addition, we noticed that the highest sensitivities (LOD of 0.6–0.7 ng/mL and 2.5–2.9 x 10^4^ CFU/mL) were obtained when the 1A4 IgG3 and 1A4 IgG3 HRP were not used in the assays (Tables 1 and 2, italics). Thus, the results demonstrate the significance of antibody subclass in assay sensitivity.

**Table 1:** Limit of detection of *F*. *tularensis* LPS (ng/mL) for each combination of 1A4 subclass variants as determined by antigen capture ELISA.

		Detector mAb		
		1A4 IgG3 HRP	1A4 IgG1 HRP	1A4 IgG2b HRP
Capture mAb	1A4 IgG3	**2.4 ± 0.23**	0.7 ± 0.05	0.9 ± 0.08
	1A4 IgG1	1.9 ± 0.37	*0*.*7* ± *0*.*09*	*0*.*7* ± *0*.*09*
	1A4 IgG2b	1.1 ± 0.32	*0*.*7* ± *0*.*06*	*0*.*6* ± *0*.*07*

The ELISAs were performed in quadruplicate and the LODs shown are mean ± standard deviation. The bold value highlights the poorest LOD, which was derived using mAb 1A4 IgG3 as both capture and detector mAbs. The italicized values highlight the improved LODs, which were obtained using non-IgG3 subclass variants of the 1A4 mAb.

**Table 2:** Limit of detection of whole killed-cells *F*. *tularensis* (10^4^ CFU/mL) for each combination of 1A4 subclass variants as determined by antigen capture ELISA.

		Detector mAb		
		1A4 IgG3 HRP	1A4 IgG1 HRP	1A4 IgG2b HRP
Capture mAb	1A4 IgG3	**10.2 ± 0.43**	3.4 ± 0.12	5.3 ± 0.30
	1A4 IgG1	6.0 ± 1.44	*2*.*5 ± 0*.*33*	*2*.*7 ± 0*.*31*
	1A4 IgG2b	3.8 ± 0.68	*2*.*6 ± 0*.*42*	*2*.*9 ± 0*.*45*

The ELISAs were performed in quadruplicate and the LODs shown are mean ± standard deviation. The bold value highlights the poorest LOD, which was derived using mAb 1A4 IgG3 as both capture and detector mAbs. The italicized values highlight the improved LODs, which were obtained using non-IgG3 subclass variants of the 1A4 mAb.

### mAb 1A4-LPS binding affinity

**Fig 3:**
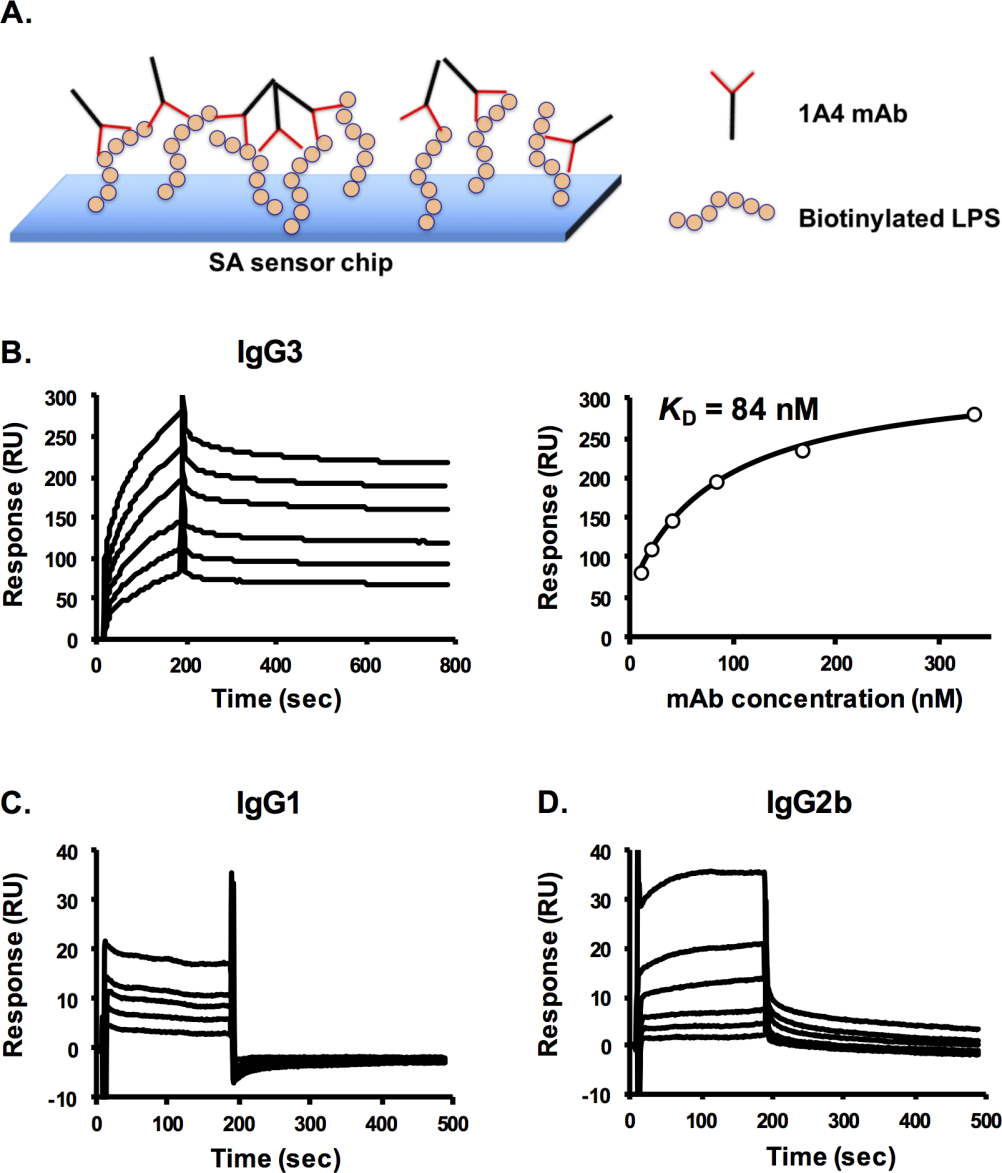
SPR analysis of LPS binding of mAbs 1A4 IgG3, IgG1 and IgG2b using a biotinylated LPS-streptavidin capture platform. Biotinylated LPS was immobilized on a streptavidin (SA) sensor chip. Sensorgrams were generated by injecting different concentrations of mAbs 1A4 IgG3 (10.4–333 nM), 1A4 IgG1(1.7–8 μM) or 1A4 IgG2b (0.1–3.3 μM) over the chip surface. Data shown is representative of three independent experiments with similar results. **Panel A,** a diagram depicting the antigen-antibody complex (and a proposed antibody-antibody interaction) formed on the chip surface. **Panel B** presents the sensorgram profile of 1A4 IgG3 (**left**) and the corresponding steady-state affinity determination (**right**). The sensorgrams for 1A4 IgG1 and IgG2b are presented in **Panels C and D**, respectively.

To understand the contribution of antibody subclass switching to assay sensitivity, the binding affinity of each 1A4 subclass variant to LPS was determined using SPR. The first SPR experiments were performed with biotinylated LPS immobilized on the SA chip surface. Fig 3A illustrates the binding complex that forms between biotinylated LPS and mAb in this assay format. The sensorgrams and steady-state binding analysis of mAb 1A4 IgG3 are presented in Fig 3B. The results showed that 1A4 IgG3 bound to immobilized LPS with a steady-state affinity (*K*_D_) of 84 nM. Analysis of 1A4 IgG1 and IgG2b showed a dramatic change in binding as a result of subclass switching (Fig 3C and 3D). There was a decrease in overall binding of the 1A4 IgG1 and IgG2b mAbs and they dissociated from the biotinylated LPS much faster than 1A4 IgG3. In this assay format, we were unable to determine the *K*_D_ values for the IgG1 and IgG2b, since the assay would have required extraordinarily high concentrations of the mAbs. Nevertheless, the results suggested that the LPS binding affinities of 1A4 IgG1 and IgG2b were substantially decreased from the parental IgG3 mAb.

**Fig 4:**
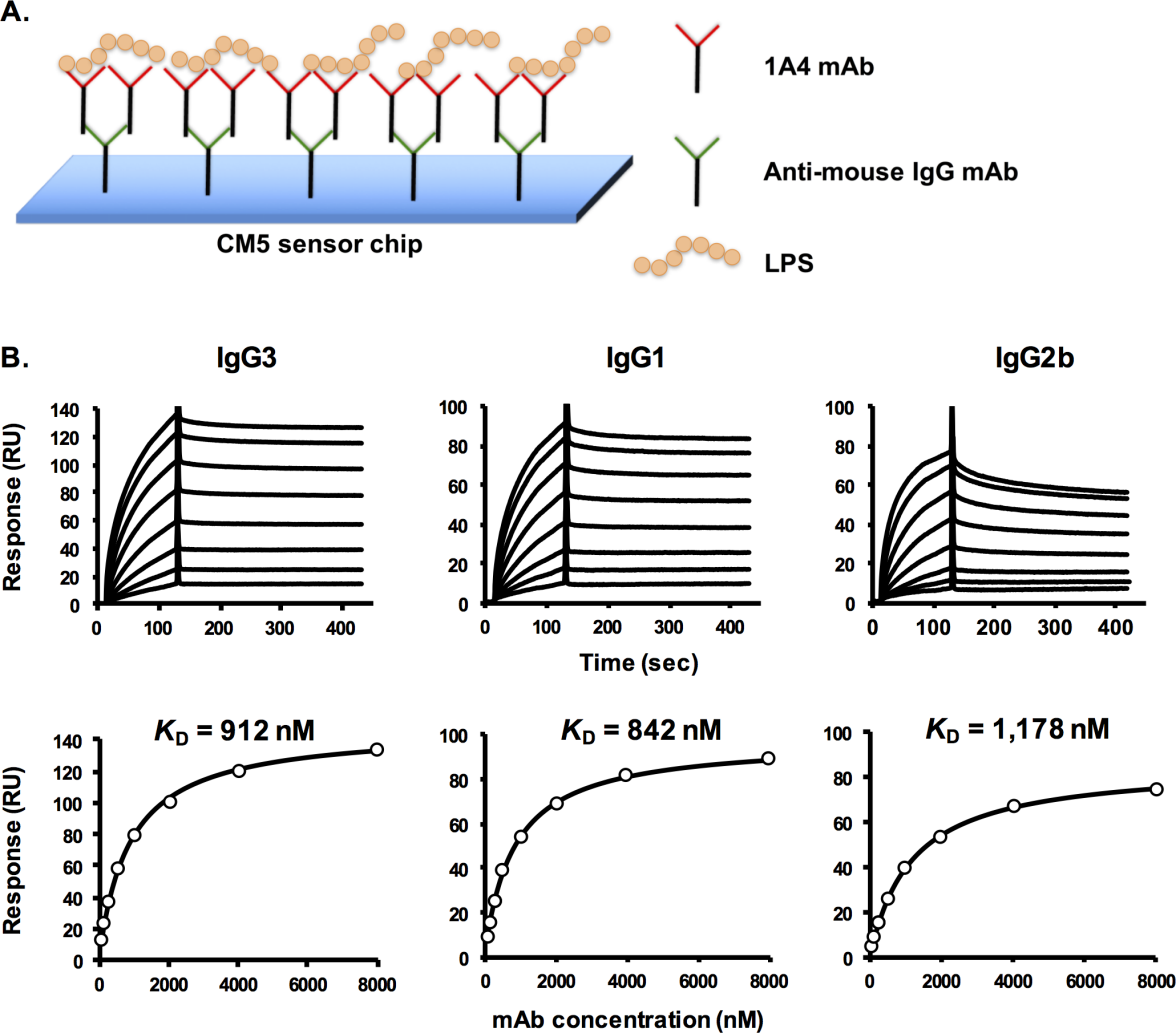
LPS binding affinity of each 1A4 subclass mAb analyzed by SPR using an antibody capture analysis approach. Anti-mouse antibody was covalently immobilized on a CM5 sensor chip. Each subclass of mAb 1A4 was injected individually over the chip surface, followed by injection of various concentrations of LPS (60–8,000 nM). Data shown is representative of three independent experiments with similar results. **Panel A** illustrates the complex formed on the chip surface. **Panel B** presents the sensorgrams (**top**) and steady-state binding analysis (**bottom**) of each 1A4 subclass variant.

Another SPR assay configuration was employed for studying LPS-mAb 1A4 binding affinity. In the second assay format, each subclass of mAb 1A4 was captured on the sensor chip surface by anti-mouse antibody immobilized on a CM5 chip. Sensorgrams were generated by injecting different concentrations of LPS over the chip surface (Fig 4B, top panel). Fig 4A depicts the binding complex formed between captured mAb 1A4 and LPS in this assay format. The *K*_D_ values for LPS binding of each 1A4 subclass mAb were determined using a steady-state affinity model (Fig 4B, bottom panel). In contrast with the previous assay, this format allowed for determination of the binding affinities for all 1A4 subclasses. The *K*_D_ values for 1A4 IgG3, IgG1 and IgG2b are 912, 842 and 1,178 nM, respectively.

**Fig 5:**
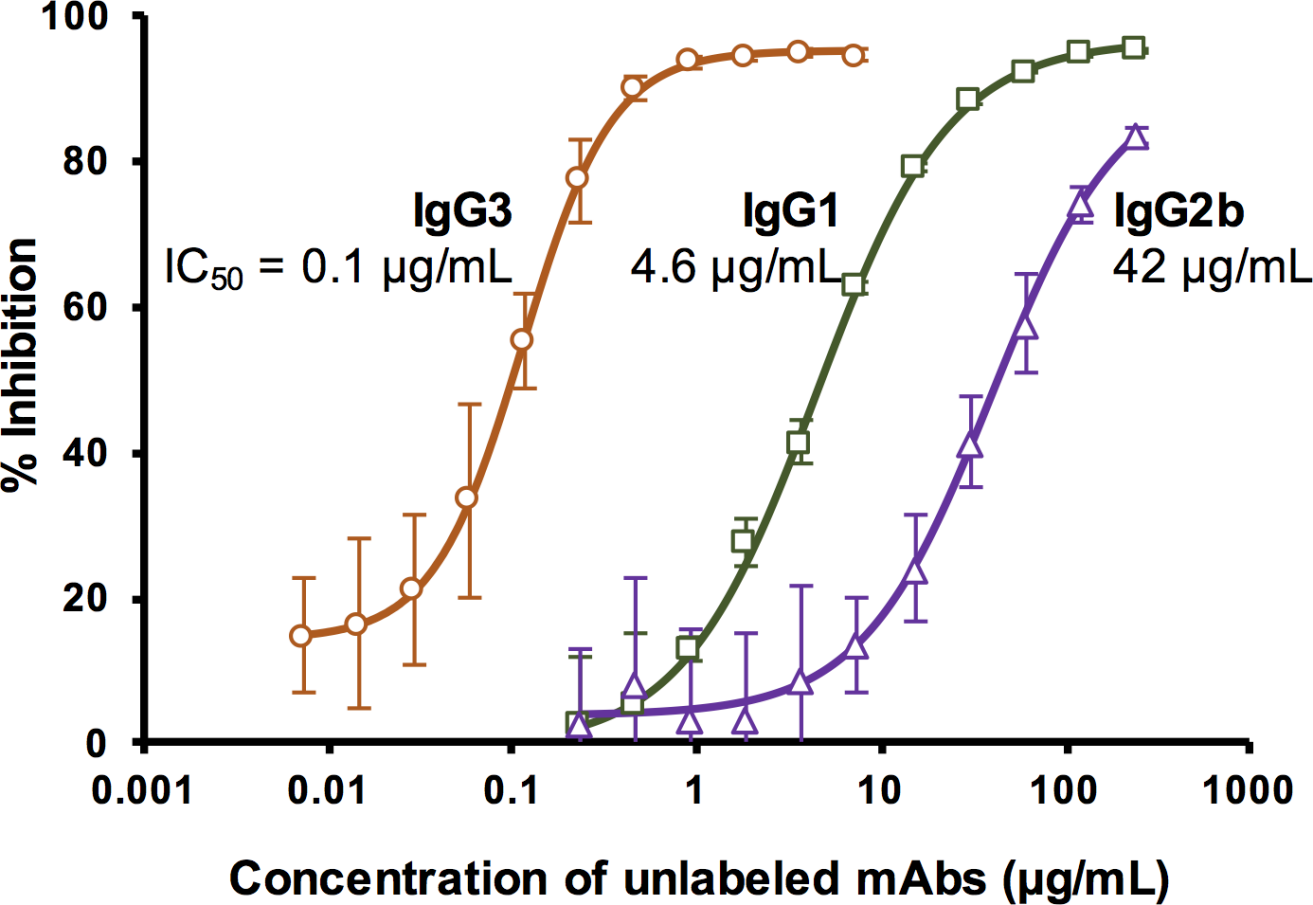
Competition ELISA for LPS binding of 1A4 subclass variants. Each 1A4 subclass mAb was assessed for its ability to compete with 1A4 IgG1 HRP conjugate for binding to LPS. Dose-response curve (%inhibition *vs* mAb concentration) of each 1A4 subclass variant was created by mixing HRP-labeled 1A4 IgG1 (fixed concentration) with various concentrations of unlabeled mAb (1A4 IgG3, IgG1 or IgG2b) before adding to microtiter plates pre-coated with LPS. Percent inhibition was calculated by % inhibition = [(OD_450_ of 1A4 IgG1 HRP alone–OD_450_ of 1A4 IgG1 HRP plus unlabeled mAb)/ OD_450_ of 1A4 IgG1 HRP alone] x 100. The analysis was performed in quadruplicate and data shown are mean ± standard deviation. The IC_50_ values were calculated from the sigmoidal dose-response curve model using SigmaPlot software.

Competition ELISA was also used to study LPS binding of 1A4 subclass variants. In this assay, microtiter plates were coated with a low concentration of LPS and a saturating concentration of HRP-labeled 1A4 IgG1 was used. This assay was designed to estimate the binding affinity of unlabeled mAbs. When mixed with HRP-labeled 1A4 IgG1, unlabeled mAb will compete with the HRP conjugate for binding to LPS, resulting in a reduction in OD_450_ (compared to 1A4 IgG1 HRP conjugate alone). Binding strength of each unlabeled mAb is then determined by its ability to outcompete 1A4 IgG1 HRP and is reported as the IC_50_, or the concentration of unlabeled mAb that inhibits the binding of 1A4 IgG1 HRP conjugate by half. Competition ELISA results for 1A4 IgG3, IgG1 and IgG2b are shown in Fig 5. As demonstrated by the IC_50_ values, LPS binding affinity of the 1A4 subclass variants are as follows: IgG3 >> IgG1 > IgG2b.

### Antibody-antibody interaction

**Fig 6:**
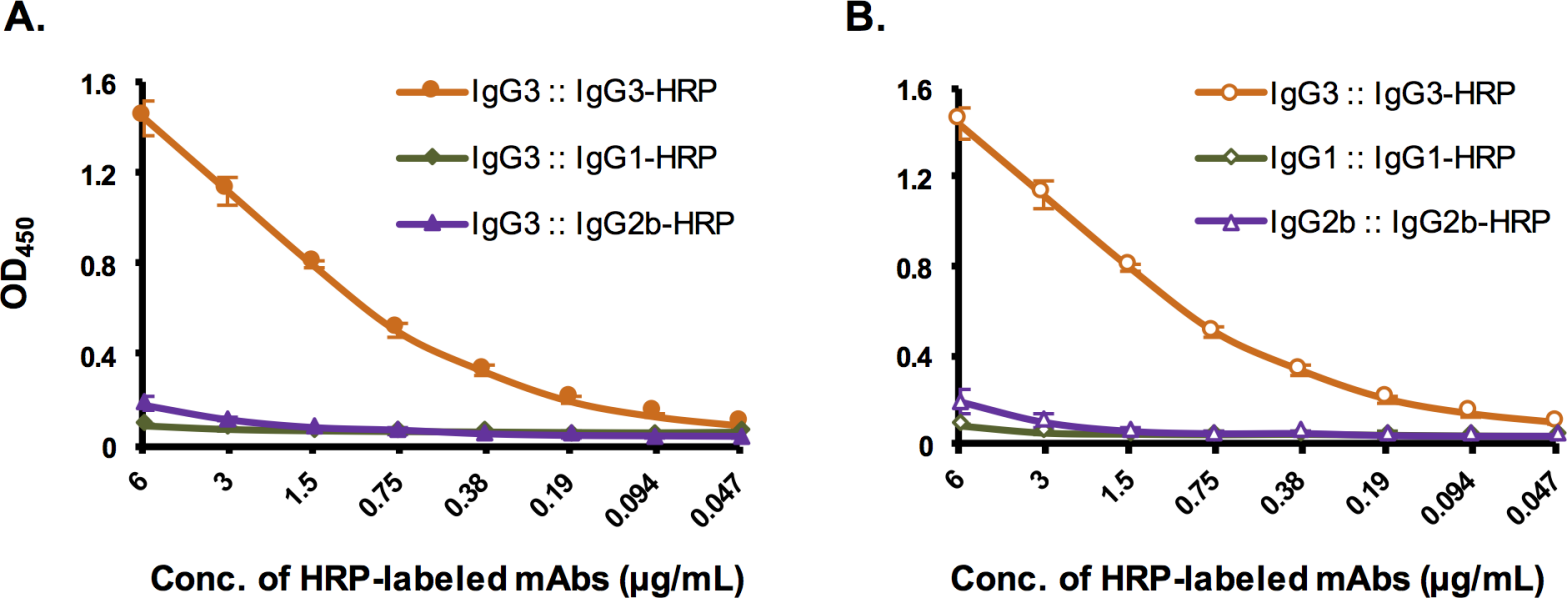
Antibody-antibody interaction as determined by direct ELISA. **Panel A**, the binding interactions between 1A4 IgG3 and each mAb subclass are demonstrated. **Panel B**, self-association of each subclass of the mAb 1A4 are shown. The experiments were carried out in quadruplicate. Data shown are mean ± standard deviation.

Antibody-antibody interactions were investigated using a direct ELISA. It was observed that 1A4 IgG3 interacted with 1A4 IgG3 itself, but no significant interaction of the IgG3 with IgG1 or IgG2b was observed (Fig 6A). We investigated further whether or not the other 1A4 subclasses might exhibit some self-association like IgG3. The results revealed that unlike the IgG3, there were no self-association with either 1A4 IgG1 or IgG2b (Fig 6B).

## Discussion

An *F*. *tularensis* LPS-specific mAb was isolated in order to develop a rapid POC diagnostic for tularemia. mAb 1A4 IgG3 was generated from a mouse immunized with whole inactivated LVS bacteria (subsp. *holarctica*). Western blot demonstrated that mAb 1A4 interacted specifically with LPS from both subsp. *tularensis* and *holarctica* without cross-reactivity with subsp. *novicida* or other species of Gram-negative bacteria tested in the study (*F*. *philomiragia*, *B*. *pseudomallei* and *E*. *coli*, Fig 1). These observations were found to be consistent with previous studies revealing that LPS from subsp. *tularensis* is identical with LPS of subsp. *holarctica*, but distinct from LPS of subsp. *novicida* [32,33]. The lack of reactivity with the lysate from an *O*-antigen mutant strain of *F*. *tularensis* (SCHU S4 ΔwbtI, Fig 1) suggested further that mAb 1A4 recognized the *O*-antigen moiety. Since the *F*. *tularensis O*-antigen is present not only in the LPS structure but also in the bacterial capsule (known as *O*-antigen capsule) [34], an immunoassay utilizing mAb 1A4 would likely be able to detect both the bacterial LPS and capsule circulating in clinical samples. Additionally, since subsp. *tularensis* and *holarctica* are responsible for the majority of human tularemia, using mAb 1A4 in an immunodiagnostic would allow for detection of most tularemia cases in a clinical setting [2]. Nevertheless, it is also important to note that there are several manifestations of tularemia, ranging from the less severe but more common ulceroglandular form to life-threatening forms of pneumonic tularemia and septicemia [2]. While the antigen-capture immunoassay is perhaps more suitable to detect severe forms of the disease (by detecting LPS in serum samples), the capability of the assay to detect the most common forms of tularemia (e.g. ulceroglandular) is possibly more limited. In the latter case, suitable methods of sample collection and/or preparation may need to be investigated to allow for detection of the antigen directly from lesions [19].

An antigen-capture immunoassay (sandwich ELISA) format was chosen for initial mAb characterization and selection of antibodies to potentially be employed in a POC diagnostic, such as a lateral flow immunoassay (LFI) [35]. The first antigen capture ELISA was developed using mAb 1A4 IgG3 as both capture and detector mAbs. The assay had a low sensitivity with a LOD of 2.4 ng/mL or 1.0 x 10^5^ CFU/mL, which is about 5-times lower than the sensitivity of a LPS-targeting antigen capture ELISA developed previously [36].

It is well established that the binding affinity of a mAb has a great impact on the sensitivity of an immunoassay [20]. Binding affinity of a mAb is defined by its antigen binding sites located in the variable domains, and traditionally thought to be unaffected by the constant domains of a mAb (except for IgA and IgM that form a dimer and pentamer, respectively). However, there are a number of recent studies demonstrating that different subclasses of IgG bearing the same antigen binding sites exhibit different binding affinity, suggesting a contribution of the constant domains to the overall binding profile [21–25]. Therefore, we investigated whether or not IgG subclass switching of 1A4 could yield a mAb with an improved LPS-binding affinity, and in turn increase sensitivity of the immunoassay.

In this study, IgG1 and IgG2b subclasses of mAb 1A4 were isolated from the parental IgG3 cell line. Unfortunately, 1A4 IgG2a could not be isolated because the hybridoma clone was unstable. LODs of the assays developed using different combinations of 1A4 subclass variants were determined (Tables 1 and 2). The results indicated that the LODs were improved when the 1A4 IgG3 HRP conjugate was replaced by 1A4 IgG1 HRP or IgG2b HRP. This finding revealed the contribution of the constant regions of IgG to the assay sensitivity.

To further investigate whether or not the increased assay sensitivity was a consequence of improved LPS-binding affinity of mAb 1A4, the affinities of each subclass variant of the mAb were assessed using SPR. In the first SPR assay configuration, the analysis was performed using the SA sensor chip coated with biotinylated LPS and with 1A4 subclass variants injected over the chip surface (Fig 3). The results showed that 1A4 IgG3 mAb has a *K*_D_ of 84 nM (Fig 3B), which is comparable to the binding affinity of other *F*. *tularensis* LPS-specific mAbs and other polysaccharide-binding mAbs described previously [19,37,38]. With this SPR assay configuration, we were unable to determine the *K*_D_ values of 1A4 IgG1 and IgG2b since the assay required abnormally high concentrations of the mAbs. Additionally, the sensorgrams demonstrated that 1A4 IgG1 and IgG2b did not bind well and dissociated from the LPS surface much faster than the IgG3 (Fig 3B and 3C). Altogether, the results suggested that the LPS-binding affinities of the IgG1 and IgG2b were substantially decreased from the parental mAb 1A4 IgG3. To confirm this result, a competition ELISA was performed (Fig 5). As demonstrated by the resultant IC_50_ values, 1A4 IgG3 (IC_50_ = 0.1 μg/mL) bound to LPS stronger than IgG1 and IgG2b (IC_50_ = 4.6 and 42 μg/mL, respectively), consistent with the SPR results.

Interestingly, it was determined that binding of all three subclasses of mAb 1A4 were very similar when the SPR analysis was performed using the antibody capture assay configuration (Fig 4). In addition, with this configuration, we also found that the *K*_D_ values of all three subclasses were comparable (Fig 4). To understand the differences in the binding affinities derived from the two different SPR antibody configurations, first we noted that the affinities determined by these assays are, in fact, a ‘functional affinity’ (or avidity) in which the bivalent binding of mAbs and all other possible interactions are taken into account. This is distinct from ‘intrinsic affinity’ in which only the interaction between one antigen binding site and a single epitope is measured. Thus, we proposed the diagrams depicting the antigen-antibody complex formed during the SPR analysis (Figs 3A and 4A). As illustrated in Fig 4A, the antibody capture configuration is likely to measure the multivalent binding between the LPS *O*-antigen epitopes and the antigen binding sites of the captured mAb. Since they are captured by the anti-mouse antibody, the self-association of mAbs 1A4 via constant regions are unlikely to occur (Fig 4A). In contrast, in the biotinylated LPS capture assay configuration, both bivalent binding (between the antigen and antibody) and potential antibody-antibody interaction (via constant regions) are taken into account (Fig 3A). Thus, it is possible that the interactions of the fragment crystallizable (Fc) regions are responsible for the decrease of the binding affinity seen in Fig 3. Additionally, since the interactions between the constant regions did not occur with the second assay configuration, the measured *K*_D_ values were not affected and remained essentially unchanged for all three subclasses of mAb 1A4 (Fig 4). In summary, these results demonstrated that the subclass switch decreased the functional affinity of mAb 1A4. The increase in assay sensitivity, which was found to be a result of the subclass switch, was clearly not because of improved mAb binding affinity to LPS as we had initially hypothesized.

How subclass switch enhances the sensitivity of the immunoassay was further investigated by the assessment of antibody-antibody interaction using a direct ELISA (Fig 6). The results show that mAb 1A4 IgG3 interacted with itself (self-association), but did not interact with other subclasses of the mAb. In addition, the 1A4 IgG1 and IgG2b did not exhibit any apparent self-association. These results support those of the antigen capture ELISA results, in that the assay background was found to be highest when mAbs 1A4 IgG3 and 1A4 IgG3 HRP were used in combination in the assay (S2 and S3 Tables). In contrast, when mAb 1A4 IgG3 was switched to IgG1 or IgG2b, the self-association was eliminated, which consequently reduced the assay background and improved the sensitivity of the assay. The results demonstrated that antibody subclass switching improved assay sensitivity through the reduction of assay background by eliminating the self-association observed with mAb 1A4 IgG3.

Notably, in this study we report the effect of IgG subclass on the functional affinity of mAb 1A4. We found that the IgG3 subclass of *F*. *tularensis* LPS mAb 1A4 exhibited the greatest functional affinity. Switching from the IgG3 to IgG1 or IgG2b subclass variants resulted in a remarkable reduction in the functional affinity. We acknowledge that our finding was inconsistent with a previous study, which demonstrated that the IgG3 subclass of a *Cryptococcus neoformans* glucuronoxylomannan (GXM) mAb showed the poorest functional affinity among the four subclasses [24]. However, our results were found to agree well with many other studies, including mAbs targeting streptococcal group A carbohydrate, LPS of *Pseudomonas aeroginosa*, poly-γ-D glutamic acid (γDPGA) capsule of *Bacillus anthracis*, and capsular polysaccharide (CPS) of *B*. *pseudomallei*. In those studies, murine IgG3 was shown to have the greatest binding affinity compared to the other subclasses [22,23,25,27]. One of the explanations for an increased functional affinity of murine IgG3 is mAb self-association, which was first described by Greenspan and Cooper [39]. In our study, self-association of mAb 1A4 IgG3 was also observed, corresponding with the previous study done by Cooper et al. [21]. Thus, it was deduced that the self-association phenomenon was one of the reasons for an increased functional affinity of mAb 1A4 IgG3.

Another consideration here is the potential for LPS itself to aggregate into supramolecular structures, sometimes with irregular shapes and molecular masses exceeding 1 x 10^6^ Daltons [40]. This phenomenon could have an impact on mAb binding, either by making LPS epitopes inaccessible (e.g. steric hindrance), or by revealing previously hidden binding sites. At very high mAb concentrations, the formation of large mAb:LPS complexes could produce unusual assay results. Antibody binding to aggregates of LPS warrants further study, as these structures may be of diagnostic and clinical relevance.

Additionally, the study indicates that it may be advisable to avoid IgG3 mAbs for the development of some immunoassays. However, murine IgG3 is the predominant subclass in response to thymus-independent (TI) antigens like LPS or CPS [41]. The ideal strategy for mAb isolation in the development of immunoassays targeting TI antigens is to use immunization strategies (such as glycoconjugates) that elicit a high affinity non-IgG3 response; however, these strategies still cannot guarantee success [41]. The results of our study suggest that subclass switching is an alternative method for enhancing the performance of immunoassays.

In conclusion, this study reports that IgG subclass selection contributes to the improved sensitivity of an immunoassay for *F*. *tularensis* LPS. Like many other studies, we observed the dramatic reduction in functional affinity of *F*. *tularensis* LPS mAb 1A4 as a result of subclass switching. However, the decreased functional affinities of IgG1 and IgG2b did not impact deteriorate overall assay performance. On the contrary, we observed an improvement in assay sensitivity when mAb 1A4 IgG1 or IgG2b was used instead of the parental mAb 1A4 IgG3. The improved assay sensitivity was due to a decrease in assay background, a result of reduced self-association of the IgG1 and IgG2b compared to the IgG3.

## Supporting information

S1 Table Concentrations of capture and detector mAbs used in the antigen capture ELISAs. (DOCX)

S2 Table Background reading (OD_450_ in the absence of purified LPS) used for calculation of LOD (ng/mL) presented in Table 1. (DOCX)

S3 Table Background reading (OD_450_ in the absence of inactivated *F*. *tularensis*) used for calculation of LOD (CFU/mL) presented in Table 2. (DOCX)

S1 ARRIVE The ARRIVE guidelines checklist for animal research/in vivo experiments. (PDF)
