# Supplementary material for: Immunoglobulin G subclass switching impacts sensitivity of an immunoassay targeting Francisella tularensis lipopolysaccharide
Source: PLoS One. 2018 Apr 9;13(4):e0195308. doi: 10.1371/journal.pone.0195308 (PMC5890998; doi:10.1371/journal.pone.0195308)
Supplement: S1 Table — (DOCX) [file pone.0195308.s001.docx]

**S1 Table. Concentrations of capture and detector mAbs used in the antigen capture ELISAs.**

| **Capture mAb : Detector mAb** | | **Concentrations** | |
| --- | --- | --- | --- |
| 1A4 IgG3 : | 1A4 IgG3 HRP | 2 µg/mL : | 0.1 µg/mL |
| 1A4 IgG3 : | 1A4 IgG1 HRP | 2 µg/mL : | 1 µg/mL |
| 1A4 IgG3 : | 1A4 IgG2b HRP | 2 µg/mL : | 1 µg/mL |
| 1A4 IgG1 : | 1A4 IgG3 HRP | 1 µg/mL : | 0.2 µg/mL |
| 1A4 IgG1 : | 1A4 IgG1 HRP | 2 µg/mL : | 1 µg/mL |
| 1A4 IgG1 : | 1A4 IgG2b HRP | 2 µg/mL : | 1 µg/mL |
| 1A4 IgG2b : | 1A4 IgG3 HRP | 2 µg/mL : | 0.5 µg/mL |
| 1A4 IgG2b : | 1A4 IgG1 HRP | 4 µg/mL : | 1 µg/mL |
| 1A4 IgG2b : | 1A4 IgG2b HRP | 4 µg/mL : | 1 µg/mL |
