# Supplementary material for: Immunoglobulin G subclass switching impacts sensitivity of an immunoassay targeting Francisella tularensis lipopolysaccharide
Source: PLoS One. 2018 Apr 9;13(4):e0195308. doi: 10.1371/journal.pone.0195308 (PMC5890998; doi:10.1371/journal.pone.0195308)
Supplement: S2 Table — (DOCX) [file pone.0195308.s002.docx]

**S2 Table. Background reading (OD_450_ in the absence of purified LPS) used for calculation of LOD (ng/mL) presented in Table 1.**

|  |  | Detector mAb | | |
| --- | --- | --- | --- | --- |
|  |  | 1A4 IgG3 HRP | 1A4 IgG1 HRP | 1A4 IgG2b HRP |
| Capture mAb | 1A4 IgG3 | 0.180 | 0.049 | 0.064 |
|  | 1A4 IgG1 | 0.185 | 0.042 | 0.045 |
|  | 1A4 IgG2b | 0.064 | 0.052 | 0.064 |
